# Herbivore-induced sorghum volatiles mediate attraction of *Cotesia flavipes*: roles of DMNT and (Z)-3-hexenyl acetate in tritrophic chemical communication

**DOI:** 10.1080/15592324.2026.2737761

**Published:** 2026-09-23

**Authors:** Cyprian Osinde, Clement Nyakoojo, Anthony M. Nsubuga, Arthur K. Tugume, Jamilu Edrisa Ssenku

**Affiliations:** a Faculty of Agriculture and Environmental Sciences, Muni University Arua, Arua, Uganda; b Department of Plant Sciences, Microbiology and Biotechnology, College of Natural Sciences, Makerere University Kampala, Kampala, Uganda; c Department of Biological Sciences Mountains of the Moon University, Fort Portal, Uganda

**Keywords:** Sorghum (*Sorghum bicolor*), herbivore-induced plant volatiles (HIPVs), *Cotesia flavipes*, DMNT, (Z)-3-hexenyl acetate, parasitoid preference, integrated pest management (IPM)

## Abstract

Herbivore-induced plant volatiles (HIPVs) influence chemical communication among plants, herbivores, and natural enemies and contribute to indirect plant defense. Previous work by our group characterized volatile emissions from sorghum following simulated herbivory and identified DMNT and (Z)-3-hexenyl acetate among consistently induced compounds, but their behavioral activity toward the stem-borer parasitoid *Cotesia flavipes* was not determined. Four-week-old sorghum (*Sorghum bicolor* L. Moench) plants were mechanically wounded and treated with *Chilo partellus* larvae oral secretions. Head-space volatile were analyzed by GC–MS, and female *C. flavipes* were tested in Y-tube olfactometer assays using volatile extracts from induced and non-damaged plants and their two-component blend. At 24 h, mean emissions of DMNT, (Z)-3-hexenyl acetate, (Z)-3-hexen-1-ol, and linalool were numerically higher in induced plants than controls; because replicate-level inferential statistics were unavailable, these differences are interpreted descriptively. In Y-tube assays, 28/40 (70.0%) females selected DMNT over hexane (χ^2^ = 6.40, df = 1, *P* = 0.011), 30/40 (75.0%) selected (Z)-3-hexenyl acetate (χ^2^ = 10.00, df = 1, *P* = 0.002), and 35/42 (83.3%) selected their blend (χ^2^ = 18.67, df = 1, *P* < 0.001). Females also preferred induced-plant extracts, with 33/45 (73.3%) selecting them over non-damaged extracts (χ^2^ = 9.80, df = 1, *P* = 0.002). These findings demonstrate laboratory behavioral activity of candidate sorghum HIPVs toward *C. flavipes*. However, they do not establish field sufficiency, concentration-dependent responses, or synergistic interactions. DMNT and (Z)-3-hexenyl acetate therefore represent candidate semiochemicals warranting further validation under ecologically realistic conditions.

## Introduction

Sorghum is a drought-adapted cereal crop of major importance in semi-arid regions, where it supports food security and livestock production. In eastern Africa, its ability to withstand drought and grow under relatively low-fertility conditions makes it particularly important in smallholder farming systems. However, sorghum production is constrained by several lepidopteran stem borers, particularly *Busseola fusca*, *Chilo partellus*, and *Sesamia calamistis*, which cause yield losses through stem tunneling, dead-heart formation, plant lodging, and impaired grain filling.^1-4^ Although sorghum is relatively resilient to several abiotic stresses, insect herbivory remains an important constraint to productivity and grain yield.^5-7^

Management of cereal stem borers has traditionally relied heavily on insecticides. However, chemical control can be ineffective against stem-borer larvae because they feed within plant tissues, where they are protected from direct exposure. Consequently, sustainable management increasingly emphasizes host-plant resistance and conservation biological control as complementary components of integrated pest management (IPM) program.^2^ ^,^ ^3^ ^,^ ^8^ ^,^ ^9^ An additional opportunity is to exploit plant traits that enhance the recruitment of parasitoids and predators. Such plant-mediated recruitment is an important component of indirect defense and may provide complementary targets for crop improvement.^10-12^ Sorghum varieties that combine direct resistance to herbivores with enhanced capacity to produce natural-enemy-attracting cues could therefore complement conventional resistance breeding and conservation biological control. However, the effectiveness of volatile-mediated recruitment must ultimately be demonstrated under field conditions.

Plants possess coordinated defense systems that enable them to perceive herbivore attack and activate interconnected biochemical and molecular signaling networks. In sorghum, herbivore perception can trigger calcium-dependent signaling, oxidative responses, and phytohormonal regulation, collectively influencing defensive metabolites and volatile organic compounds (VOCs).^13-17^ These responses can affect herbivore performance and interactions with organisms at higher trophic levels. Among them, herbivore-induced plant volatiles (HIPVs) are particularly important because they function as airborne signals in interactions among plants, herbivores, and natural enemies. HIPVs comprise several chemical classes, including terpenoids, green leaf volatiles, aromatic compounds, and nitrogen-containing metabolites, and can transmit information within plant communities and across trophic levels.^18-20^ By attracting predators and parasitoids, these compounds can contribute to indirect plant defense and potentially influence the biological suppression of herbivores.^18-21^

Sorghum provides a useful system for investigating such HIPV-mediated interactions. Zhuang et al.^22^ demonstrated dynamic herbivore-induced sesquiterpene biosynthesis in sorghum and related grasses, while Park et al.^23^ showed that sorghum volatile profiles can discriminate plants subjected to sugarcane aphid infestation. More recently, Russavage et al.^24^ demonstrated cultivar-dependent variation in aphid-induced volatiles and associated preference of natural enemies in sorghum. Together, these studies indicate that sorghum volatile responses are influenced by herbivory and genotype and can mediate interactions with organisms at higher trophic levels.

Parasitoids exploit herbivore-associated changes in plant odor as indirect indicators of potentially suitable host habitats. By detecting volatile signatures associated with herbivore presence or feeding damage, parasitoids can improve host-searching efficiency. The composition and relative abundance of induced volatiles vary with plant species, genotype, herbivore identity, and feeding mode, potentially providing parasitoids with information that facilitates discrimination among host-associated habitats.^18^ ^,^ ^20^ ^,^ ^25^ Consequently, the biological activity of a HIPV cannot be inferred solely from its presence in a plant head-space, behavioral assays are required to establish whether parasitoids detect and use individual compounds or odor blends during host location.

Among HIPVs reported from sorghum and other cereal systems, the homoterpene 4,8-dimethyl-1,3,7-nonatriene (DMNT) and the green leaf volatile (Z)-3-hexenyl acetate are of particular interest as candidate semiochemicals. DMNT is produced through herbivore-induced terpenoid biosynthetic pathways and has been implicated in the preference or orientation of parasitoids and predators in several agricultural systems, including maize, cotton, and rice.^20^ Similarly, (Z)-3-hexenyl acetate is generated through the lipoxygenase pathway following tissue damage and forms part of the rapidly emitted green leaf volatile response associated with herbivory and mechanical injury.^26^ ^,^ ^27^ Several parasitoids, including *Cotesia marginiventris*, *Microplitis croceipes*, and *Cotesia flavipes*, exploit herbivore-associated plant odors during host-searching behavior.^28-30^ Importantly, parasitoid responses can depend not only on the total amount of volatile material released but also on the identity, relative abundance, and combination of compounds within an odor blend.^31-33^ Thus, detecting a compound in an herbivore-induced headspace does not establish that it is itself a behaviorally active parasitoid cue.

Previous work by Osinde et al.^34^ characterized herbivore-induced volatile emissions from sorghum and identified DMNT and (Z)-3-hexenyl acetate among the compounds associated with simulated herbivory. However, the occurrence of these compounds in the sorghum volatile profile does not by itself establish their ecological or behavioral function. In particular, it remained unknown whether these individual compounds are detected by the stem-borer parasitoid *Cotesia flavipes* and whether they can influence its odor-guided orientation. Thus, an important gap remained between the chemical characterization of herbivore-induced sorghum volatiles and their functional significance in tritrophic chemical communication.

The present study addresses this gap by testing the behavioral activity of DMNT and (Z)-3-hexenyl acetate individually and in combination in female *C. flavipes*, and by comparing parasitoid responses to volatile extracts from simulated-herbivore-induced and non-damaged sorghum using Y-tube olfactometer assays. The study therefore does not seek to identify new sorghum volatile compounds; rather, its novelty lies in providing a behavioral validation of previously characterized sorghum volatiles and examining whether these compounds are associated with orientation responses in a parasitoid of an economically important sorghum stem borer. By linking volatile occurrence with parasitoid behavior, the study provides a functional step toward understanding how herbivore-induced chemical cues may participate in sorghum–herbivore–parasitoid communication.

The biological relevance of the induction system also requires careful consideration. *Chilo partellus* is primarily an internal stem feeder, whereas the present experiment used leaf mechanical damage combined with larval oral secretions. This treatment was therefore intended as a standardized simulated-herbivory treatment rather than a direct reproduction of natural stem feeding. Mechanical wounding introduced tissue damage, while oral secretions provided herbivore-associated chemical elicitors. Because volatile responses to simulated herbivory may differ from those induced by larvae feeding within stems, the findings are interpreted as evidence of laboratory behavioral activity rather than a direct representation of field-level parasitoid recruitment.

We hypothesized that female *C. flavipes* would prefer plant volatiles such as DMNT, (Z)-3-hexenyl acetate, and their blend from induced sorghum over extracts from non-damaged plants. By integrating previous chemical characterization with controlled parasitoid behavioral assays, this study provided functional evidence for the behavioral activity of candidate sorghum HIPVs and established a basis for subsequent concentration–response, blend-interaction, and field-validation studies aimed at exploiting HIPV-mediated biological control in sorghum.

## Materials and methods

### Plant material and growth conditions

Sorghum (*Sorghum bicolor* L. Moench) cultivar Epuripur was selected based on its use in our previous volatile-emission study.^34^ Seeds were germinated under greenhouse conditions and transplanted into plastic pots containing sterilized loam soil. Soil was sterilized by autoclaving at 121 °C for 30 min and allowed to cool to room temperature before use to minimize uncontrolled variation from soil-borne microorganisms and arthropods that could influence plant defense or volatile emissions. Plants were maintained under a 12 h light/12 h dark photoperiod at 26 ± 3 °C and 70–75% relative humidity. Plants were watered daily, with the amount adjusted as necessary to maintain comparable soil moisture among treatments. Four-week-old sorghum plants with comparable height, biomass, and leaf number were selected and randomly assigned to undamaged control or herbivore-induced treatment groups. For volatile-emission analysis, four independent plants (*n* = 4) were used per treatment, with each plant representing one biological replicate. Volatile collections were performed independently for each plant, and each plant was treated as the experimental unit for statistical analysis. Technical measurements obtained from the same plant were averaged before inferential analysis and were not treated as independent biological replicates.

### Collection and rearing of *Chilo partellus* for oral-secretion collection

Larvae of *Chilo partellus* (Swinhoe) (Lepidoptera: Crambidae), the spotted stem borer, were collected from naturally infested sorghum fields. Plants showing typical stem-borer symptoms, including foliar feeding damage, dead-heart symptoms, and larval frass around stem entry points, were identified. Infested stems were carefully dissected, and larvae were removed using sterile forceps. Individual larvae were transferred to ventilated plastic containers containing fresh sorghum leaves to minimize handling stress and maintain survival during transportation to the laboratory. Similar field collection and handling procedures have been used to establish and maintain *C. partellus* populations for laboratory studies.^3^ ^,^ ^35^

Upon arrival, larvae were examined for developmental stage, physical condition, and signs of pathogen or parasitoid infestation. Individuals showing injury, disease, pupation, or parasitism were excluded. Healthy larvae were maintained individually under controlled laboratory conditions and reared on fresh sorghum leaves until the required developmental stage for oral-secretion collection. Leaves were replaced every two days to maintain food availability and minimize microbial contamination. Individual rearing prevented cannibalism and ensured that oral-secretion samples represented independent biological material.^36^

Third-instar larvae were used for oral-secretion collection because oral-secretions composition and concentration can vary with larval developmental stage. Oral secretions were collected from actively feeding third-instar larvae following gentle stimulation and pooled. Oral secretions were collected from approximately 20 individual third-instar larvae and pooled to obtain one biological sample. Approximately 1–2 µL was collected per larva, yielding approximately 20–40 µL pooled sample. Samples were transferred to sterile microcentrifuge tubes and stored at −20 °C and extracts were analyzed within one week. Before application, pooled sample secretions were thawed on ice and diluted three-fold with sterile distilled water. Oral secretions were used as herbivore-associated elicitors to induce sorghum defense responses.^37^ ^,^ ^38^

### Simulated herbivore treatment

Sorghum plants were subjected to mechanical damage followed by application of *C. partellus* oral secretions to simulate herbivore-associated damage. Oral secretions contain herbivore-associated molecular patterns (HAMPs) and elicitors capable of activating plant defense signaling beyond the effects of mechanical wounding alone.^14^ ^,^ ^37-41^

Mechanical damage was applied to three fully expanded leaves per plant using a sterile fabric pattern wheel. Each leaf received five standardized passes over the designated area, producing approximately 10 cm^2^ of wounded tissue per leaf (approximately 30 cm^2^ per plant). Immediately after wounding, 1 mL of three-fold-diluted oral secretion was uniformly applied to the wounded areas using a micropipette. The same pooled oral-secretion preparation and application volume were used for all replicate plants within the treatment. Non-damaged control plants received neither mechanical wounding nor oral secretion.

The treatment represented simulated herbivory rather than natural *C. partellus* infestation. It incorporated two experimentally controllable components of herbivore-associated damage—mechanical tissue disruption and exposure to herbivore-associated oral secretions—but did not reproduce the spatial location, feeding pattern, duration, or temporal progression of natural stem-borer feeding. Because *C. partellus* larvae predominantly feed within the stem, the responses observed here should therefore be interpreted as responses to simulated herbivory and oral-secretions treatment rather than as a direct representation of responses to naturally developing *C. partellus* infestation.

Following treatment, plants were maintained under greenhouse conditions for 24 h before volatile collection. This interval was selected as a standardized sampling point because herbivore-induced volatile organic compounds (HIPVs), including green leaf volatiles, terpenoids, and benzenoids, can accumulate rapidly following herbivore attack and contribute to direct and indirect plant defense.^18^ ^,^ ^19^ ^,^ ^29^ ^,^ ^42-44^

Because HIPV emissions are dynamic and individual compounds can differ in their induction and emission patterns following herbivore-associated damage, the 24-h sampling interval represents a time-specific assessment rather than the maximum or cumulative volatile-emission capacity of the plants.^45^

### Collection and analysis of sorghum volatile organic compounds

#### Dynamic headspace sampling

Volatile organic compounds (VOCs) were collected from individual treated and control sorghum plants using dynamic head space sampling, a widely used approach for capturing herbivore-induced plant volatiles under controlled conditions.^46-48^ The aerial portion of each plant was enclosed in a clean, inert glass chamber measuring approximately 30 × 30 × 50 cm. The chamber was fitted with airtight inlet and outlet ports connected by inert Teflon tubing, and direct contact between plant tissues and chamber walls was avoided. Before each collection, the chamber and tubing were thoroughly cleaned and flushed with purified air to minimize background contamination.

Purified, charcoal-filtered air was continuously passed through the chamber using a vacuum pump at approximately 500 mL min^−1^, while plant-emitted VOCs were simultaneously withdrawn at approximately 500 mL min^−1^ through a Super Q adsorbent trap containing approximately 30–50 mg of adsorbent.^46^ ^,^ ^48^ ^,^ ^49^

Volatile collections were conducted for 4 h during the light period, when emissions of many green leaf volatiles and terpenoids are influenced by light and circadian regulation.^18^ ^,^ ^19^ ^,^ ^50^ ^,^ ^51^ An empty-chamber control was collected using the same chamber, tubing, airflow rates, trap type, and collection duration to identify potential background contaminants from the chamber, tubing, adsorbent, or incoming air.

Following collection, Super Q traps were eluted with 1.0 mL analytical-grade hexane containing a known concentration of *n*-nonane as the internal standard. The eluates were transferred to clean, solvent-resistant glass vials, tightly sealed, and stored at −20 °C until GC–MS analysis. The internal standard was added at a constant concentration to all samples and blank extracts to facilitate semi-quantitative determination of volatile compounds and account for variation in extraction and instrumental response. Empty-chamber controls underwent the same extraction procedure.

#### Gas chromatography–mass spectrometry analysis

VOCs collected from induced and control plants were identified and quantified using gas chromatography–mass spectrometry (GC–MS), following established methods for plant volatile analysis.^47^ ^,^ ^52^ VOC extracts were analyzed using a Shimadzu GCMS-QP2010 SE gas chromatograph–mass spectrometer (Shimadzu Corporation, Kyoto, Japan) equipped with an Rtx-5MS capillary column (30 m × 0.25 mm internal diameter × 0.25 µm film thickness). Helium (99.9% purity) was used as the carrier gas at approximately 1.0 mL min^−1^. The oven temperature was held at 40 °C for 2 min, increased at 5 °C min^−1^ to 150 °C, then at 10 °C min^−1^ to 250 °C, and held for 5 min. Injector and transfer-line temperatures were 250 °C. The mass spectrometer operated in electron-ionization mode at 70 eV, acquiring spectra over m/z 40–400.

Compound identification was based on mass-spectral comparison with the NIST/EPA/NIH Mass Spectral Library (NIST 20), retention indices, and authentic reference standards where available.^47^ Particular attention was given to herbivore-associated VOCs, including DMNT [(E)-4,8-dimethyl-1,3,7-nonatriene], (Z)-3-hexenyl acetate, other green leaf volatiles, and terpenoids.^14^ ^,^ ^18^ ^,^ ^19^ ^,^ ^29^ ^,^ ^43^ ^,^ ^44^ ^,^ ^46^ ^,^ ^53^

DMNT and (Z)-3-hexenyl acetate were confirmed using authentic analytical standards (≥98% purity; Sigma-Aldrich/Merck, Germany). Sample retention times and calculated retention indices were compared with those of the corresponding standards analyzed under identical GC–MS conditions, with confirmation further supported by matching mass spectra with NIST 20 reference spectra. Thus, compound identification was based on the combined agreement of retention time, retention index, and mass-spectral characteristics rather than library similarity alone. These compounds were also identified in our previous experiment.^34^

Volatile emissions were quantified relative to the internal standard and expressed as ng g^−1^ fresh weight h^−1^, following established normalization procedures.^47^ ^,^ ^54^ Fresh weight was determined immediately after VOC collection by weighing the leaves enclosed within the collection chamber using a calibrated analytical balance. The same leaf-sampling procedure was applied across treatments to ensure consistent normalization of VOC emissions.

Natural volatile extracts were chemically characterized by GC–MS, whereas the behavioral blend assay used a defined synthetic mixture of DMNT and (Z)-3-hexenyl acetate. No multivariate or interaction analysis of the natural volatile blend was performed.

### Preparation of volatile samples for behavioral assays

Volatile extracts from simulated-herbivore-induced and non-damaged plants were obtained from the Super Q traps using the same solvent used for chemical analysis. Extracts were transferred into amber glass vials fitted with PTFE-lined caps to minimize volatilization, oxidation, and photodegradation and stored at −20 °C.^47^ ^,^ ^49^

Immediately after elution, extracts were divided into small aliquots to minimize repeated freeze–thaw cycles and potential changes in volatile composition during storage.^46^ ^,^ ^47^ ^,^ ^55^

Before behavioral assays, stored extracts were allowed to equilibrate to room temperature and gently mixed to ensure homogeneity. A defined volume of 10 µL of VOC aliquot representing a fraction, corresponding to the extract obtained from 1.0 g fresh weight of sampled sorghum leaves, was applied evenly to Whatman No. 1 filter paper and allowed to equilibrate for approximately 30–60 s before placement in the appropriate arm of the Y-tube olfactometer. This procedure minimized residual-solvent exposure while providing a standardized volatile stimulus.^11^ ^,^ ^48^ ^,^ ^56^

The 10-µL aliquot provided a standardized quantity of VOC extract for behavioral testing rather than a defined concentration of individual compounds. The volume corresponding to 1.0 g fresh weight was calculated from the total extract volume and the fresh weight represented by each VOC collection. The amounts of principal compounds in the corresponding extracts, particularly DMNT and (Z)-3-hexenyl acetate, were determined by GC–MS. Equivalent extract volumes were used across replicates, and solvent-only extracts were processed identically as controls.

### Parasitoid rearing

The parasitoid used in the behavioral assays was *Cotesia flavipes* Cameron (Hymenoptera: Braconidae), a gregarious larval endoparasitoid and an important biological control agent of lepidopteran stem borers in cereal crops, particularly *Chilo partellus* and other economically important stem-borer species in Africa and Asia.^57-59^ *Cotesia flavipes* was obtained from a laboratory colony maintained at the International Center of Insect Physiology and Ecology (ICIPE), Nairobi, Kenya. The colony was maintained at 25 ± 2 °C, 70 ± 10% relative humidity, and a 12 h light/12 h dark photoperiod.

Third-instar *C. partellus* larvae were used as hosts for parasitoid rearing based on their documented suitability for development of *C. flavipes.* ^58^ Adult parasitoids were supplied with 10% (v/v) honey solution as a carbohydrate source to support adult survival and longevity, following established parasitoid-rearing practices.^60^ ^,^ ^61^

Adult female *C. flavipes* used in behavioral assays were sexually mature and host-naïve, having had no previous opportunity to encounter or oviposit on *C. partellus* larvae before testing. Females were provided access to males for mating before behavioral assays and were subsequently maintained under the same laboratory conditions until use. Thus, females tested in the Y-tube assays were mated but oviposition-naïve.

### Y-tube olfactometer bioassays

Behavioral responses were evaluated using a custom-built two-arm glass Y-tube olfactometer consisting of a 15-cm- long central release stem connected to two 15-cm- long symmetrical arms, with an internal diameter of 1.5 cm leading to separate odor-source chambers, connected at 60 ° angle at the choice point. The Y-tube approach is widely used to investigate olfactory responses of parasitoids and other insects to plant-derived volatile cues.^11^ ^,^ ^32^ ^,^ ^46^ ^,^ ^56^

Purified, charcoal-filtered, and humidified air was passed through each arm into the respective odor chamber. Airflow was maintained at approximately 200 mL min^−1^ per arm, giving a total airflow of approximately 400 mL min^−1^. Odor sources were presented simultaneously at the Y-junction, and their positions were regularly alternated between the two arms. Airflow was maintained at approximately 200 mL min^−1^ per arm to provide consistent odor delivery. Odor-source positions were alternated between arms across assays, and treatment order was randomized where applicable to minimize positional bias.^47-49^ ^,^ ^56^

Bioassays were conducted under diffuse laboratory illumination of approximately 300–500 lux, with the olfactometer positioned on a vibration-free bench and shielded from direct visual disturbance. Individual female parasitoids were introduced into the release arm and allowed 5 minutes to respond. A choice was recorded when a parasitoid entered an odor arm beyond the predetermined decision point, approximately 5 cm beyond the Y-junction, and remained there for at least 30 seconds.^32^ ^,^ ^46^ ^,^ ^56^ Females that did not enter either arm within 5 minutes were recorded as no-choice responses and excluded from preference analyzes, consistent with established olfactometer protocols.^11^ ^,^ ^33^ ^,^ ^62^

Between assays, the olfactometer, odor-source chambers, and connecting components were cleaned with 70% ethanol followed by distilled water and allowed to dry completely. Arm positions and odor-source locations were alternated regularly to minimize positional and residual-odor effects.^20^ ^,^ ^32^ ^,^ ^47^ ^,^ ^56^ Each female was tested only once to avoid pseudoreplication and effects of previous exposure.

### Experimental odor treatments based on identified HIPVs

Behavioral treatments were selected based on HIPVs identified in sorghum. Four pairwise comparisons were evaluated from herbivore-induced sorghum and non-damaged sorghum. These included: (i) hexane solvent versus DMNT, (ii) hexane solvent versus (Z)-3-hexenyl acetate, (iii) hexane solvent versus a DMNT + (Z)-3-hexenyl acetate blend, (iv) hexane solvent Vs VOCs from non-induced sorghum.

For individual-compound treatments, DMNT or (Z)-3-hexenyl acetate was applied at 10 µg per filter-paper strip. For the blend treatment, DMNT and (Z)-3-hexenyl acetate were each applied at 10 µg per strip, giving a total of 20 µg per blend. Because only one concentration was tested for each treatment, these assays assessed parasitoid preference to standardized chemical stimuli and did not evaluate concentration-dependent behavioral responses.

### Statistical analysis

For volatile-emission measurements, the individual plant was treated as the experimental unit. Volatile emissions were summarized as mean ± standard deviation (SD), with the number of independent biological replicates reported as *n = 4* for each treatment. Because replicate-level inferential comparisons were not available, differences in volatile emissions were interpreted descriptively rather than as statistically tested treatment effects. Statistical significance was evaluated at *α* = 0.05.

For parasitoid behavioral assays, the individual female *Cotesia flavipes* was treated as the experimental unit. For each assay, the total number of females tested (*N*), number making a choice (*n*), number selecting each odor source, and number making no choice were recorded. No-choice individuals were excluded from the binary preference analysis but were retained in reporting of the total number tested. Preference was expressed as the proportion of responding females selecting the treatment odor.

The four odor comparisons were analyzed as separate two-choice behavioral assays: DMNT versus hexane, (Z)-3-hexenyl acetate versus hexane, the DMNT+(Z)-3-hexenyl acetate blend versus hexane, and volatile extract from non-damaged sorghum versus hexane. For each assay, the numbers of females selecting each odor source were compared with an expected 1:1 distribution using a chi-square goodness-of-fit test. Degrees of freedom were 1 for all comparisons, and the corresponding χ^2^ statistic and two-sided *P*-value were reported. A *P*-value < 0.05 was considered significant. Percent preference was calculated as the proportion of responding females that selected the treatment odor.

Because the four behavioral assays represented separate two-choice experiments, their preference percentages were not treated as directly comparable treatment means, and no statistical inference regarding differences among DMNT, (Z)-3-hexenyl acetate, the blend, and induced-plant extracts was made from their respective percentages. Furthermore, each chemical treatment was tested at a single concentration, and the individual compounds and their blend were not arranged in a factorial treatment structure. Consequently, the data were not used to estimate statistical interaction effects between DMNT and (Z)-3-hexenyl acetate. The response to the blend was therefore interpreted as enhanced preference relative to the individual treatments only in a descriptive sense and not as statistical evidence of synergism, antagonism, or additive.

### Ethical and biosafety considerations

All experiments (insect colonies, plant material, and laboratory procedures) conducted followed institutional biosafety guidelines and relevant regulatory requirements.

## Results

### Herbivore-associated changes in sorghum volatile emissions

The present analysis focused quantitatively on four compounds detected in the sorghum headspace: the homoterpene DMNT [(E)-4,8-dimethyl-1,3,7-nonatriene], the green leaf volatiles (Z)-3-hexenyl acetate, (Z)-3-hexen-1-ol blend and the monoterpene linalool. Volatile emissions are presented as mean ± SD of four independent biological replicates.

At 24 h after simulated herbivory, DMNT emission was 2.76 ± 0.31 ng g^−1^ FW h^−1^ (*n* = 4) in simulated-herbivore-induced plants compared with 0.85 ± 0.12 ng g^−1^ FW h^−1^ (*n* = 4) in non-damaged controls, corresponding to a 3.2-fold difference. Similarly, (Z)-3-hexenyl acetate emission was 3.45 ± 0.42 ng g^−1^ FW h^−1^ (*n* = 4) in induced plants compared with 1.10 ± 0.18 ng g^−1^ FW h^−1^ (*n* = 4) in controls, corresponding to a 3.1-fold difference. Emission of (Z)-3-hexen-1-ol was 5.10 ± 0.62 ng g^−1^ FW h^−1^ (*n* = 4) in induced plants compared with 2.30 ± 0.40 ng g^−1^ FW h^−1^ (*n* = 4) in controls, representing an approximately 2.2-fold difference. Linalool emission was 1.12 ± 0.15 ng g^−1^ FW h^−1^ (*n* = 4) in induced plants and 0.45 ± 0.08 ng g^−1^ FW h^−1^ (*n* = 4) in non-damaged controls, corresponding to an approximately 2.5-fold difference. All these results are presented in Table 1.

**Table 1.** Volatile emission profile of non-damaged and simulated-herbivore-induced sorghum plants.

Compound	Non-damaged control mean ± SD (ng g^−1^ FW h^−1^)	Simulated-herbivore-induced mean ± SD (ng g^−1^ FW h^−1^)	Fold change
DMNT	0.85 ± 0.12	2.76 ± 0.31	3.2
(Z)-3-Hexenyl acetate	1.10 ± 0.18	3.45 ± 0.42	3.1
(Z)-3-Hexen-1-ol	2.30 ± 0.40	5.10 ± 0.62	2.2
Linalool	0.45 ± 0.08	1.12 ± 0.15	2.5

Note: values are mean ± SD of independent biological replicates (*n* = 4) plants per treatment. Fold change is the ratio of mean volatile emission in simulated-herbivore-induced plants to that in non-damaged controls. The four compounds shown were selected for quantitative comparison and do not represent an exhaustive inventory of all compounds detected in the headspace.

These values describe the magnitude and variability of volatile emissions at the 24-h sampling point. Because replicate-level data and corresponding inferential statistical analyzes were not available for these VOC measurements, no *P*-values or claims of statistical significance are assigned to these treatment differences. The observed differences were therefore interpreted as descriptive evidence of altered volatile emission following simulated herbivory rather than as statistically demonstrated treatment effects.

### Parasitoid response to individual HIPVs

Female *Cotesia flavipes* showed significant preferences for both tested individual HIPVs relative to hexane. In the DMNT assay, 45 females were tested, of which 40 made a choice and five made no choice. Among responding females, 28/40 (70.0%) selected DMNT, whereas 12/40 (30.0%) selected hexane. The distribution of choices differed significantly from the expected 1:1 ratio (χ^2^ = 6.40, df = 1, *P* = 0.011).

In the (Z)-3-hexenyl acetate assay, 45 females were tested, with 40 responding and five making no choice. Among responding females, 30/40 (75.0%) selected (Z)-3-hexenyl acetate, compared with 10/40 (25.0%) selecting hexane. The observed distribution differed significantly from the expected 1:1 ratio (χ^2^ = 10.00, df = 1, *P* = 0.002). These results are presented in Table 2.

**Table 2.** Y-tube olfactometer responses of *Cotesia flavipes* females to sorghum volatile stimuli.

Odor comparison	Treatment choice, *n*	Control choice, *n*	No choice, *n*	Total tested, *N*	Preference (%)	χ^2^	df	*P*-value
DMNT vs hexane	28	12	5	45	70.0	6.40	1	0.011
(Z)-3-Hexenyl acetate vs hexane	30	10	5	45	75.0	10.00	1	0.002
DMNT+(Z)-3-hexenyl acetate vs hexane	35	7	3	45	83.3	18.67	1	<0.001
Induced sorghum vs non-damaged sorghum	33	12	5	50	73.3	9.80	1	0.002

Note: *N* = total number of females tested; *n* = number selecting the indicated odor source. Preference (%) was calculated as treatment choices/(treatment choices + control choices) × 100 and therefore excludes no-choice individuals. No-choice responses were recorded but excluded from the 1:1 preference tests. χ^2^ values are from chi-square goodness-of-fit tests against an expected 1:1 distribution; df = 1.

These findings demonstrate that both DMNT and (Z)-3-hexenyl acetate were behaviorally active at the single concentrations tested. However, the results do not establish concentration-dependent preference, behavioral thresholds, or that one compound is intrinsically more important than the other.

### Response to the DMNT+(Z)-3-hexenyl acetate blend

The DMNT+(Z)-3-hexenyl acetate blend elicited the strongest numerical response among the tested chemical stimuli. Of 45 females tested, 42 made a choice and three made no choice. Among responding females, 35/42 (83.3%) selected the blend, whereas 7/42 (16.7%) selected hexane. The distribution differed significantly from the expected 1:1 ratio (χ^2^ = 18.67, df = 1, *P* < 0.001) as presented in Table 2.

The high proportion of females selecting the blend demonstrates that the combined chemical stimulus was attractive under the experimental conditions. However, because the study used a single concentration for each constituent and did not employ a factorial design, this result does not provide a statistical test of interaction, synergy, or antagonism between DMNT and (Z)-3-hexenyl acetate. The response is therefore interpreted as strong preference for the combined stimulus rather than evidence of synergism.

### Preference for simulated-herbivore-induced sorghum volatiles

Female *C. flavipes* also preferred volatile extracts from simulated-herbivore-induced sorghum over extracts from non-damaged plants. Of 50 females tested, 45 made a choice and five made no choice. Among responding females, 33/45 (73.3%) selected the simulated-herbivore-induced sorghum extract, whereas 12/45 (26.7%) selected the non-damaged sorghum extract. The distribution differed significantly from the expected 1:1 ratio (χ^2^ = 9.80, df = 1, *P* = 0.002) as presented in Table 2.

This finding demonstrates that volatile extracts from simulated-herbivore-induced sorghum contained olfactory information capable of influencing parasitoid orientation under the experimental conditions. Because these extracts contained multiple volatile constituents, the preference cannot be attributed specifically to DMNT, (Z)-3-hexenyl acetate, or any other individual compound.

### Summary of parasitoid preference

Among responding females, the highest preference was observed for the DMNT + (Z)-3-hexenyl acetate blend (35/42, 83.3%), followed by (Z)-3-hexenyl acetate (30/40, 75.0%), simulated-herbivore-induced sorghum volatile extract (33/45, 73.3%), and DMNT (28/40, 70.0%). All four odor comparisons showed significant deviations from the expected 1:1 distribution (P ≤ 0.011). These percentages describe the proportion of responding females choosing the treatment odor and should not be interpreted as statistically significant differences among the four preference percentages.

## Discussion

### Behavioral validation of sorghum herbivore-induced volatiles

The principal finding of this study is that selected volatile compounds associated with herbivore-induced sorghum emissions elicited orientation responses in female *C. flavipes*. This finding provides a behavioral extension of our previous volatile-characterization study,^34^ in which DMNT and (Z)-3-hexenyl acetate were identified among compounds associated with simulated-herbivore-induced sorghum. Whereas the previous study established their occurrence in the induced volatile profile, the present study demonstrates that these compounds can influence the orientation of a parasitoid relevant to Sorghum stem-borer biological control in the laboratory.

This distinction is important because the detection or induction of a volatile compound does not by itself establish behavioral function. Behavioral assays provide an additional level of evidence by determining whether natural enemies respond to the compound or blend under controlled conditions.

The observed parasitoid preferences are consistent with the established role of HIPVs in tritrophic chemical communication. Herbivore-associated plant odors can provide information used by parasitoids during host-location behavior.^11^ ^,^ ^29^ ^,^ ^30^ ^,^ ^33^ Herbivore-associated damage can activate plant defense signaling and increase production of green leaf volatiles and terpenoid compounds that contribute to indirect plant defense.^18^ ^,^ ^20^ ^,^ ^44^ ^,^ ^52^ These volatile signals may allow natural enemies to distinguish damaged from undamaged plants and thereby improve the efficiency of host searching.^19^ ^,^ ^43^ ^,^ ^53^

The present findings therefore strengthen the link between sorghum volatile induction and parasitoid behavior by demonstrating that the chemical changes observed following simulated herbivory were accompanied by measurable behavioral responses from *C. flavipes*.

### DMNT and (Z)-3-hexenyl acetate as candidate parasitoid-attracting cues

Female *C. flavipes* significantly preferred both DMNT and (Z)-3-hexenyl acetate over hexane at the concentrations tested. DMNT was selected by 28/40 responding females (70.0%; χ^2^ = 6.40, df = 1, *P* = 0.011), whereas (Z)-3-hexenyl acetate was selected by 30/40 responding females (75.0%; χ^2^ = 10.00, *P* = 0.002). The blend elicited an even higher numerical proportion of choices (35/42, 83.3%; χ^2^ = 18.67, *P* < 0.001). However, these percentages were obtained from separate two-choice assays and were not statistically compared with one another. Consequently, the numerically greater response to the blend should not be interpreted as evidence that the blend was significantly more attractive than either individual compound.

Similarly, the preference for simulated-herbivore-induced sorghum extracts was statistically different from a 1:1 distribution, with 33/45 responding females (73.3%) selecting induced-plant extracts over non-damaged-plant extracts (χ^2^ = 9.80, *P* = 0.002). This result demonstrates behavioral discrimination between the two odor sources under the assay conditions but does not identify the specific volatile constituents responsible for the response.

DMNT is a widely distributed herbivore-induced homoterpene associated with herbivore-activated terpenoid metabolism, whereas (Z)-3-hexenyl acetate is a green leaf volatile commonly associated with tissue disruption and herbivore attack.^18^ ^,^ ^20^ ^,^ ^26^ ^,^ ^52^

The behavioral activity of these compounds is consistent with previous studies showing that parasitoids can use individual HIPVs as olfactory cues during host searching.^11^ ^,^ ^28-30^ Green leaf volatiles can be released rapidly following tissue damage, while terpenoid-derived signals may provide additional information about herbivore-associated plant status.^27^ ^,^ ^43^

The present results identify DMNT and (Z)-3-hexenyl acetate as candidate chemical cues capable of influencing *C. flavipes* orientation. However, the results should be interpreted specifically within the concentration and assay conditions used here. Because only one concentration of each compound was tested, the study cannot determine behavioral thresholds, optimal concentrations, or concentration-dependent changes in preference.

The numerically greater response to (Z)-3-hexenyl acetate than to DMNT should therefore not be interpreted as evidence that (Z)-3-hexenyl acetate is a stronger or more important ecological cue. Such a comparison would require an appropriately designed experiment with standardized dose–response relationships and, ideally, direct statistical comparison between compounds.

### Response to the combined HIPV treatment

The DMNT+(Z)-3-hexenyl acetate blend generated the highest numerical preference among the synthetic odor treatments, with 83.3% of responding females selecting the blend. This result indicates that the combined stimulus was strongly attractive under the experimental conditions and is consistent with the broader concept that parasitoids can use combinations of volatile compounds during host location.^11^ ^,^ ^33^ ^,^ ^62^

However, the present design does not permit a formal test of synergism. The compounds were not independently varied in a factorial design, and only one concentration was evaluated for each compound. Consequently, the higher response to the blend cannot be statistically partitioned into independent, additive, synergistic, or antagonistic effects.

The appropriate interpretation is therefore that the blend produced the highest numerical preference among the tested stimuli, rather than that DMNT and (Z)-3-hexenyl acetate acted synergistically.

This distinction is particularly important because parasitoids may respond to the relative composition of volatile blends rather than simply to the presence or absolute quantity of individual compounds. Characteristic combinations of HIPVs may provide more reliable information about herbivore-associated plants than isolated constituents.^11^ ^,^ ^20^ ^,^ ^31^

### Preference for herbivore-induced sorghum volatile extracts

Female *C. flavipes* preferred extracts from simulated-herbivore-induced sorghum over extracts from non-damaged plants. This response provides complementary evidence to the blended-compound assays because it demonstrates that the parasitoids responded not only to individual candidate HIPVs but also to the complex volatile mixture produced by sorghum following simulated herbivory.

The result is consistent with the established framework of tritrophic chemical communication, whereby herbivore-induced plants release volatile blends that can attract natural enemies.^18-20^ Comparable responses have been documented in maize, cotton, rice, and other crop systems, where parasitoids orient toward herbivore-associated plant odors during host searching.^11^ ^,^ ^29^ ^,^ ^53^

Importantly, the induced-versus-non-damaged plant comparison cannot establish which compound or combination of compounds was responsible for the observed preference. The induced extracts contained multiple volatile constituents, including DMNT, (Z)-3-hexenyl acetate, (Z)-3-hexen-1-ol, and linalool. Therefore, the observed response should be attributed to the altered volatile blend as a whole rather than to a specific constituent.

### Integration of chemical and behavioral evidence

The combination of volatile profiling and Y-tube bioassays provides two complementary levels of evidence. First, mean emissions were higher in simulated-herbivore-induced plants of DMNT, (Z)-3-hexenyl acetate, (Z)-3-hexen-1-ol, and linalool at the 24-h sampling point. Second, two of the identified compounds, individually and in combination, elicited significant orientation responses from *C. flavipes*. The preference for induced-plant extracts further demonstrates that the overall altered volatile profile was behaviorally active.

This progression from chemical characterization to behavioral validation represents the principal contribution of the present study. The compounds themselves are not newly discovered sorghum volatiles; rather, the study provides behavioral evidence that DMNT and (Z)-3-hexenyl acetate, previously associated with herbivore-induced sorghum emissions,^34^ can influence the orientation of *C. flavipes.Therefore, t*he novelty of the present study lies not in identifying new sorghum volatiles, but in providing experimentally controlled behavioral evidence that two previously characterized sorghum HIPVs are detected and preferentially selected by female *C. flavipes*, thereby linking sorghum volatile production with parasitoid orientation

Such behavioral validation is important because volatile abundance alone cannot establish ecological relevance. A compound may increase following herbivory without being detected by a natural enemy or without eliciting a behavioral response. Conversely, Compounds present at relatively low abundance can, in some systems, contribute disproportionately to information content if they are behaviorally active^33^ ^,^ ^62^; however, the present study did not test this relationship directly.

The present findings therefore support the designation of DMNT and (Z)-3-hexenyl acetate as candidate parasitoid-attracting HIPVs in sorghum, while recognizing that their ecological importance remains to be established under natural infestation and field conditions.

### Biological interpretation of simulated herbivory

The simulated-herbivory treatment combined mechanical tissue damage with application of *C. partellus* oral secretions. This approach provides experimental standardization and incorporates both physical injury and herbivore-associated elicitors, which can activate defense responses beyond those induced by mechanical damage alone.^14^ ^,^ ^37-41^

Nevertheless, the treatment did not reproduce natural *C. partellus* feeding. Larvae of this species predominantly feed within sorghum stems, whereas the present treatment was applied to leaves. Natural infestation can additionally involve sustained feeding, larvae movement, frass deposition, vascular damage, and prolonged herbivore-associated signaling. These differences could influence both the magnitude and composition of volatile emissions.

Accordingly, the present findings should be interpreted as evidence that standardized simulated herbivory can induce parasitoid-relevant volatile cues, rather than as a direct characterization of the volatile plume generated by naturally infested sorghum.

### Temporal considerations in volatile emission

The volatile measurements were obtained 24 h after simulated herbivory. HIPV emissions are dynamic, and individual compounds can exhibit distinct induction and emission kinetics following herbivore-associated damage.^45^ ^,^ ^50^ ^,^ ^51^ Therefore, the concentrations reported here represent a specific temporal snapshot rather than the maximum or cumulative volatile-emission capacity of sorghum.

This temporal limitation is particularly relevant when interpreting the relationship between volatile emission and parasitoid behavior. A compound that is highly induced at one time point may not remain abundant throughout the period during which parasitoids search for hosts. Conversely, compounds with rapid induction may function as early indicators of herbivore attack.

Time-course experiments examining volatile emission and parasitoid behavior at multiple post-induction intervals would therefore be valuable for determining whether the behavioral activity of DMNT and (Z)-3-hexenyl acetate corresponds with their temporal emission dynamics.

### Volatile quantity, blend composition, and parasitoid orientation

The results also reinforce the distinction between volatile abundance and volatile information content. Parasitoid orientation is unlikely to depend solely on the absolute amount of an individual compound. Instead, behavioral responses may depend on compound identity, concentration, relative proportions within a blend, and the ecological context in which the odor is encountered.^11^ ^,^ ^33^ ^,^ ^62^

The preference for the induced sorghum extract suggests that the parasitoids recognized information associated with the altered volatile blend. However, the synthetic-compound experiments demonstrate that at least two constituents of this blend can independently influence parasitoid orientation. These findings support a model in which individual HIPVs may provide informative cues while their combination contributes to a more complex odor signal.

### Implications for biological control

The observed preference of *C. flavipes* to DMNT, (Z)-3-hexenyl acetate, their combination, and induced-sorghum volatile extracts has potential implications for biological control of cereal stem borers. The findings identify DMNT and (Z)-3-hexenyl acetate as candidate semiochemicals for subsequent evaluation. However, laboratory preference should not be equated with field efficacy. Before these compounds can be considered for practical application, field experiments are required to determine whether their deployment increases parasitoid visitation, residence time, parasitism, and suppression of *C. partellus* populations.

The progression from chemical characterization to behavioral validation can provide a foundation for such studies. Semiochemical-based approaches have potential to complement biological control and reduce dependence on broad-spectrum insecticides while supporting ecological pest management.^9^ ^,^ ^20^

An additional implication concerns sorghum breeding. If sorghum genotypes differ consistently in their production of parasitoid-attracting HIPV blends following herbivory, volatile traits could potentially be incorporated into screening and breeding programs alongside direct resistance traits. Such an approach could contribute to integrated resistance combining plant defense with recruitment of natural enemies. The present study does not test genotypic variation and therefore provides a basis for, rather than evidence of, this potential application.

## Conclusions

This study provides behavioral evidence that DMNT and (Z)-3-hexenyl acetate, volatile compounds previously associated with herbivore-induced sorghum emissions, influence the orientation of female *Cotesia flavipes* under controlled Y-tube olfactometer conditions. Simulated herbivory was associated with increased emission of DMNT, (Z)-3-hexenyl acetate, (Z)-3-hexen-1-ol, and linalool at the 24-h sampling point. Female parasitoids preferred DMNT, (Z)-3-hexenyl acetate, their two-component blend, and volatile extracts from simulated-herbivore-induced sorghum over their respective controls.

The principal contribution of the study is behavioral validation rather than chemical discovery. The findings extend our previous characterization of sorghum herbivore-induced volatiles^34^ by demonstrating that selected compounds from the induced volatile profile can elicit orientation responses from *C. flavipes* . The response to the induced-plant extract further indicates that the broader volatile blend generated following simulated herbivory contains information capable of influencing parasitoid orientation.

The blend produced the highest numerical preference among the tested stimuli and conditions, but does not establish synergism because the experiment did not include independent concentration levels or a factorial design. Similarly, because only one concentration of each compound was tested, the results do not establish concentration-dependent behavioral responses.

DMNT and (Z)-3-hexenyl acetate can therefore be regarded as candidate HIPVs for further investigation of sorghum–parasitoid chemical communication. Future research should determine their concentration–response relationships, temporal dynamics, behavior under natural *C. partellus* infestation, and ecological effectiveness under field conditions. Such studies could ultimately establish whether HIPV-mediated recruitment of *C. flavipes* can contribute to integrated stem-borer management and whether parasitoid-attracting volatile traits can be incorporated into sorghum improvement programs.

## Limitations and future research

Several limitations should be considered when interpreting the findings.

First, the use of simulated herbivory provides standardized induction but does not reproduce natural *C. partellus* infestation. Future studies should compare naturally infested and experimentally induced plants to determine whether they produce comparable volatile profiles and parasitoid responses.

Second, volatile emissions were characterized at a single 24-h sampling point. Time-course experiments are needed to establish the induction and persistence of DMNT, (Z)-3-hexenyl acetate, and other candidate HIPVs.

Third, only one concentration of each synthetic compound was tested. Dose–response experiments should therefore be conducted to establish behavioral thresholds, optimal concentrations, and potential changes in preference at higher or lower stimulus intensities.

Fourth, the blend experiment cannot establish synergism because the individual compounds were not independently varied in a factorial design. Future experiments should manipulate DMNT and (Z)-3-hexenyl acetate independently across multiple concentrations and compare observed blend responses with a predefined additive model.

Fifth, Y-tube olfactometer assays provide controlled measurements of olfactory preference but do not reproduce the spatial and environmental complexity of an agricultural field. Wind, temperature, humidity, plant architecture, competing odors, and host-associated cues can all modify parasitoid responses under natural conditions.^11^ ^,^ ^20^

Finally, the induced plant extract contained multiple compounds, meaning that the behavioral response cannot be attributed exclusively to DMNT and (Z)-3-hexenyl acetate. Electrophysiological assays, multicomponent blend experiments, and field studies would help determine which constituents are detected by *C. flavipes* and which combinations provide the most reliable host-associated information.

Collectively, these limitations define a logical progression for future work: validation under natural stem-borer infestation, characterization of temporal and concentration-dependent responses, mechanistic analysis of volatile-blend interactions, and field testing of candidate semiochemicals.
